# Long-Term Cardiovascular Outcomes of Glucagon-Like Peptide-1 (GLP-1) Receptor Agonists in Type 2 Diabetes: A Systematic Review

**DOI:** 10.7759/cureus.73705

**Published:** 2024-11-14

**Authors:** Salman Tariq, Mirza Ahmed Ali, Hafiz Muhammad Hassan Iftikhar, Muhammad Fareh Ali, Syed Qamber Ali Shah, Fouzia Perveen, Tahir Zaman

**Affiliations:** 1 General Internal Medicine, East Lancashire Hospitals NHS Trust, Blackburn, GBR; 2 General Medicine, Bashiran Sadiq Cheema Hospital, Wazirabad, PAK; 3 Medicine, Pakistan Air Force Hospital, Islamabad, PAK; 4 Pharmacology, Nawaz Sharif Medical College, Gujrat, PAK; 5 Medicine, University Hospital Dorset, Royal Bournemouth Hospital, Dorset, GBR; 6 Pharmacology, Shalamar Medical and Dental College, Lahore, PAK; 7 General Medicine, General Hospital Lahore, Lahore, PAK

**Keywords:** cardiorenal protection, cardiovascular outcomes, comparative effectiveness, diabetes management, glp-1 receptor agonists, major adverse cardiovascular events (mace), metabolic effects, patient subgroups, systematic review, type 2 diabetes mellitus

## Abstract

Glucagon-like peptide-1 receptor agonists (GLP-1 RAs) have emerged as a promising class of medications for type 2 diabetes (T2D) management. While their glucose-lowering effects are well-established, their long-term impact on cardiovascular outcomes remains a subject of ongoing research and debate.

This systematic review aims to assess the long-term cardiovascular effects of GLP-1 RAs in adults with T2D compared to placebo, standard care, or other glucose-lowering medications. We systematically searched PubMed, Embase, and the Cochrane Library for randomized controlled trials (RCTs) and observational studies published from database inception to April 2024. Two independent reviewers screened the studies and extracted the data. The primary outcome was major adverse cardiovascular events (MACE), a composite of cardiovascular death, non-fatal myocardial infarction (MI), and non-fatal stroke. Secondary outcomes included individual components of MACE, hospitalization for heart failure, and all-cause mortality.

We included 15 studies (eight RCTs and seven observational studies) involving over 180,000 participants. GLP-1 RAs were associated with a significant reduction in MACE compared to placebo or standard care (risk ratio: 0.88, 95% CI: 0.82-0.94, p<0.001). GLP-1 RAs also demonstrated superior cardiovascular protection compared to DPP-4 inhibitors and sulfonylureas. The benefits were particularly pronounced in reducing the risk of stroke and MI. Notably, some studies found larger cardiovascular benefits in frail patients. The effects on heart failure outcomes were mixed, with potential attenuated benefits in patients with baseline heart failure. GLP-1 RAs also showed promising effects on renal outcomes and metabolic parameters. The quality of evidence ranged from moderate to high across outcomes.

This systematic review provides strong evidence that GLP-1 RAs offer significant cardiovascular benefits in adults with T2D, particularly in reducing MACE, stroke, and MI. The findings support current guidelines recommending GLP-1 RAs as preferred agents in patients with established cardiovascular disease or high cardiovascular risk. However, the variability in effects across different patient subgroups underscores the need for personalized treatment approaches. Future research should focus on head-to-head comparisons between different GLP-1 RAs, long-term follow-up studies, and investigation of combination therapies to further optimize the use of these agents in clinical practice.

## Introduction and background

Type 2 diabetes mellitus (T2DM) is a global health concern, affecting an estimated 462 million people worldwide [1]. The disease is characterized by chronic hyperglycemia and is associated with numerous complications, particularly cardiovascular disease (CVD), which remains the leading cause of morbidity and mortality in this population [2]. The management of T2DM has evolved significantly over the past two decades, with a shift from a glucose-centric approach to one that emphasizes cardiovascular risk reduction [3,4].

Glucagon-like peptide-1 receptor agonists (GLP-1 RAs) have emerged as a promising class of glucose-lowering medications with potential cardiovascular benefits. These agents mimic the action of the incretin hormone GLP-1, enhancing glucose-dependent insulin secretion, suppressing glucagon release, and slowing gastric emptying [5]. Beyond their glycemic effects, GLP-1 RAs have demonstrated favorable impacts on body weight, blood pressure, and lipid profiles, all of which are important cardiovascular risk factors [6,7].

The cardiovascular safety of diabetes medications became a focal point following concerns raised about rosiglitazone in 2007 [8]. Subsequently, regulatory agencies mandated cardiovascular outcome trials (CVOTs) for new diabetes therapies [9]. This requirement has led to a wealth of data on the cardiovascular effects of various glucose-lowering agents, including GLP-1 RAs [10].

Several large-scale CVOTs have investigated the cardiovascular safety and efficacy of GLP-1 RAs. The LEADER trial, which evaluated liraglutide, was the first to demonstrate a significant reduction in major adverse cardiovascular events (MACE) compared to placebo in patients with T2DM at high cardiovascular risk. Subsequent trials, such as SUSTAIN-6, REWIND, and AMPLITUDE, have shown similar benefits with other GLP-1 RAs, albeit with some variations in the magnitude and specific components of cardiovascular risk reduction [11,12].

Despite these promising results, questions remain regarding the long-term cardiovascular effects of GLP-1 RAs. The generalizability of CVOT findings to broader patient populations, the comparative effectiveness against other glucose-lowering medications, and the potential for class effects versus agent-specific benefits are areas of ongoing investigation [13,14]. Moreover, the mechanisms underlying the cardiovascular benefits of GLP-1 RAs are not fully elucidated, with both direct effects on the cardiovascular system and indirect effects through improvement of metabolic parameters being proposed [15,16].

Recent meta-analyses have attempted to synthesize the available evidence on GLP-1 RAs and cardiovascular outcomes. A comprehensive meta-analysis by Kristensen et al. (2019) found that GLP-1 RAs reduced MACE by 12% compared to placebo, with significant reductions in cardiovascular mortality, fatal or non-fatal stroke, and fatal or non-fatal myocardial infarction (MI) [17]. A more recent network meta-analysis by Tsapas et al. (2020) confirmed these findings and provided additional insights into the relative efficacy of different GLP-1 RAs [18]. However, these analyses were limited to data from randomized controlled trials (RCTs) and did not include more recent studies or real-world evidence.

The evolving landscape of diabetes management has seen GLP-1 RAs gain prominence in clinical guidelines. The American Diabetes Association and the European Association for the Study of Diabetes now recommend GLP-1 RAs as a preferred option for patients with T2DM and established atherosclerotic CVD or high cardiovascular risk [2,3]. However, the optimal positioning of these agents in treatment algorithms, particularly with other cardioprotective glucose-lowering medications such as sodium-glucose cotransporter-2 (SGLT2) inhibitors, remains a subject of debate [19,20].

As the use of GLP-1 RAs continues to increase, there is a need for a comprehensive evaluation of their long-term cardiovascular effects, incorporating evidence from both RCTs and observational studies. This systematic review aims to assess the long-term cardiovascular outcomes associated with GLP-1 RA use in adults with T2DM, compare their cardiovascular effects with other glucose-lowering medications, and evaluate their safety profile in long-term use. By synthesizing the most up-to-date evidence, including recent trials and real-world data, this review seeks to provide clinicians and policymakers with a robust foundation for decision-making in the management of T2DM and cardiovascular risk.

Aims and objectives

Aim of the Study

This study aims to assess the long-term cardiovascular outcomes associated with GLP-1 RA use in adults with T2D.

Objectives

The objectives include the assessment of the long-term cardiovascular effects of GLP-1 RAs. Additionally, to compare the cardiovascular effects of GLP-1 RAs with other glucose-lowering medications, to evaluate the effects of GLP-1 RAs on other body systems, and finally, to evaluate the safety profile of GLP-1 RAs in long-term use.

## Review

Research question and protocol

Research Question (PICO)

In adults with T2DM, what are the long-term cardiovascular effects of GLP-1 RAs compared to placebo, standard care, or other glucose-lowering medications? The PICO framework is given in Table 1.

**Table 1:** PICO framework T2DM, type 2 diabetes mellitus; MACE, major adverse cardiovascular events; GLP-1 RAs, glucagon-like peptide-1 receptor agonists; MI, myocardial infarction

PICO	Components
Population	Adults (≥18 years) with T2DM
Intervention	GLP-1 RAs (any type, dose, or duration)
Comparison	Placebo, standard care, or other glucose-lowering medications (e.g., DPP-4 inhibitors, SGLT-2 inhibitors, sulfonylureas, or insulin)
Outcomes	Long-term cardiovascular outcomes (primary: MACE; secondary: cardiovascular death, non-fatal MI, non-fatal stroke, hospitalization for heart failure, and all-cause mortality)

​​​​​​*Protocol*

Eligibility criteria: RCTs and observational studies (cohort and case-control studies) were included. Studies include adults (≥18 years) with T2DM. Focusing on the interventions of any GLP-1 RA. The comparator groups included placebo, standard care, or other glucose-lowering medications. The outcomes focus on at least one long-term cardiovascular outcome reported in the included studies.

Information sources: Information sources included the electronic databases (PubMed, Embase, Cochrane Library) and the clinical trial registries (ClinicalTrials.gov, WHO International Clinical Trials Registry Platform).

Data management: Use of reference management software for deduplication and screening and data extraction forms developed a priori and piloted before full use.

Selection process: Two independent reviewers screened titles and abstracts, followed by a full-text review. The disagreements were resolved by consensus or the involvement of a third reviewer.

Data collection process: Two independent reviewers extracted data using standardized forms.

Data items: Study characteristics include the design, setting, and duration. Participant characteristics include age, sex, diabetes duration, and comorbidities. Intervention details include GLP-1 RA type, dose, and duration. Other include the comparison details, outcome measures, and time points. 

Outcomes and prioritization: The primary outcome is MACE (composite of cardiovascular death, non-fatal MI, and non-fatal stroke) while secondary outcomes are individual components of MACE, hospitalization for heart failure, and all-cause mortality.

Data synthesis: Narrative synthesis of findings reported along with subgroup analyses based on GLP-1 RA type, comparison group, and baseline cardiovascular risk.

Methodology

Criteria

Only English-language publications on RCTs and observational studies including prospective cohort and case-control studies were included. A population of adults (≥18 years) with T2DM and studies focusing primarily on intervention as any GLP-1 RA (e.g., liraglutide, semaglutide, dulaglutide, and exenatide) were included. Studies with comparison groups of placebo, standard care, or other glucose-lowering medications were included in this systematic review, along with the studies with outcomes of at least one long-term cardiovascular outcome reported.

Studies focusing exclusively on type 1 diabetes or gestational diabetes were excluded along with animal studies or in vitro experiments, case reports, case series, and narrative reviews. The studies do not report any of the pre-specified cardiovascular outcomes.

Search Strategy

The search strategy was developed in consultation with a medical librarian and includes a combination of Medical Subject Headings (MeSH) terms and free-text terms. The strategy will be adapted for each database.

Sample search strategy: The same strategies were developed for PubMed, Embase, and Cochrane Library.

"Glucagon-Like Peptide-1 Receptor"[Mesh] OR "glucagon-like peptide-1 receptor agonist*"[tiab] OR GLP-1[tiab] OR liraglutide[tiab] OR semaglutide[tiab] OR dulaglutide[tiab] OR exenatide[tiab]

"Diabetes Mellitus, Type 2"[Mesh] OR "type 2 diabetes"[tiab] OR T2DM[tiab]

"Cardiovascular Diseases"[Mesh] OR cardiovascular[tiab] OR "heart disease"[tiab] OR "myocardial infarction"[tiab] OR stroke[tiab] OR "heart failure"[tiab]

"Long-Term Effectiveness"[tiab] OR "long-term outcome*"[tiab] OR "long-term effect*"[tiab]

#1 AND #2 AND #3 AND #4

Filters: Human studies, English language

Study Selection

Two independent reviewers screened the titles and abstracts of all retrieved articles using the predefined inclusion and exclusion criteria. Full texts of potentially eligible studies were obtained and reviewed independently by the same two reviewers. Any disagreements were resolved through discussion or, if necessary, by involving a third reviewer. The selection process was documented using a PRISMA flow diagram.

**Figure 1:**
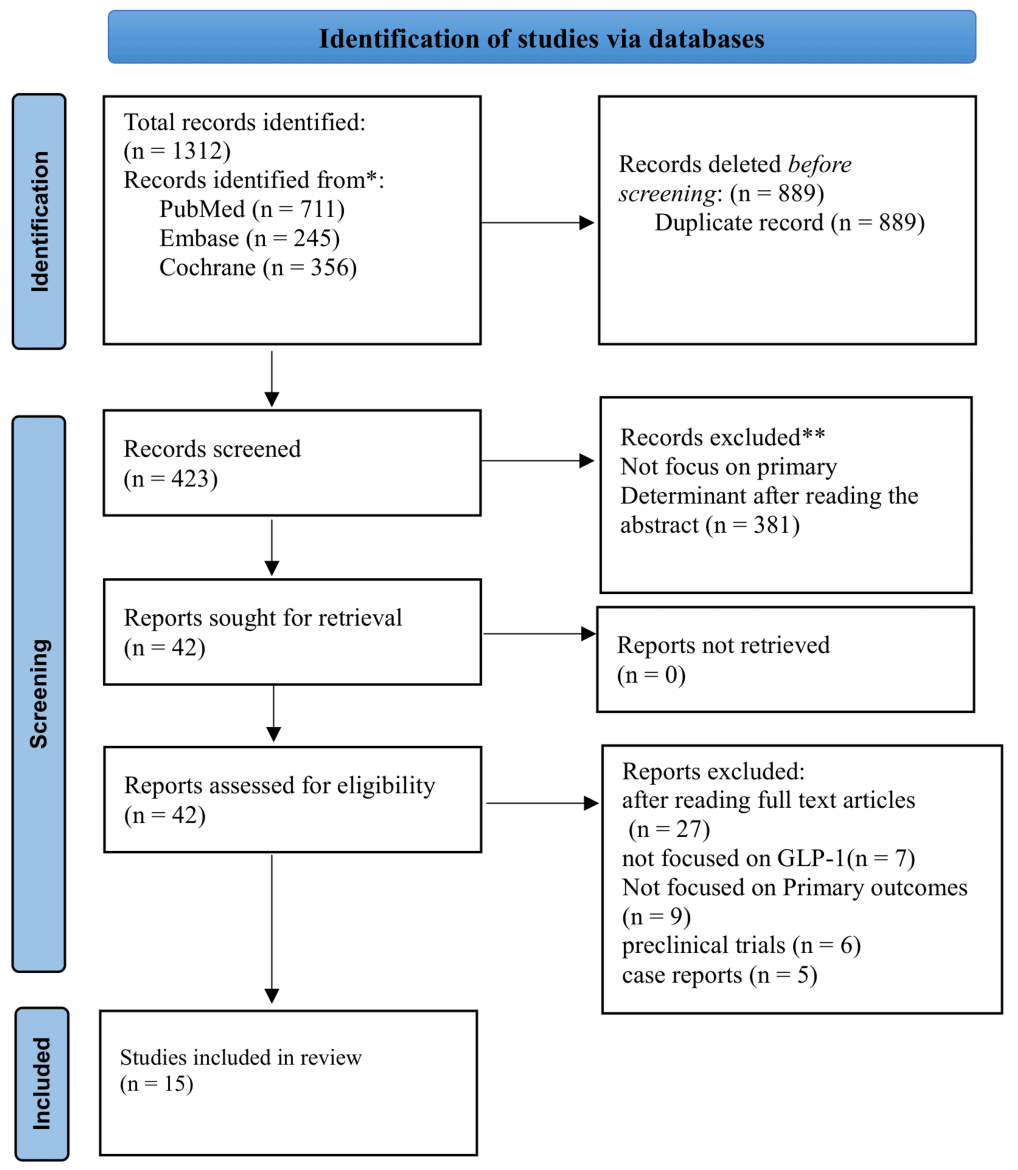
Prisma flow chart

Figure 1 shows details regarding the selection of the study. The initial search yielded 1312 studies (PubMed: 711, Embase: 245, Cochrane: 356). After removing 889 duplicates, 423 studies remained for screening. Following title and abstract screening, 381 studies were excluded based on our predefined criteria, leaving 42 studies for full-text review. Of these, 27 studies were excluded due to irrelevant outcomes and inappropriate study designs. Ultimately, 15 studies met all eligibility criteria and were included in the final analysis.

Data Extraction

A standardized, pre-piloted form was used to extract data from the included studies for assessment of study quality and evidence synthesis. Two reviewers extracted the data independently, with any discrepancies resolved by discussion or involvement of a third reviewer. The following data was extracted: study characteristics (authors, year of publication, country, study design, sample size, and follow-up duration), participant characteristics (age, sex, diabetes duration, baseline HbA1c, cardiovascular risk factors, and comorbidities), intervention details (GLP-1 RA type, dose, and duration of treatment), comparison details (type of control or comparison treatment), outcome measures (definition and timing of cardiovascular outcomes), and results (number of events, effect sizes, confidence intervals, p-values).

Outcome Measures

Primary outcome: MACE: A composite of cardiovascular death, non-fatal MI, and non-fatal stroke.

Secondary outcomes: Individual components of MACE (cardiovascular death, non-fatal MI, and non-fatal stroke), hospitalization for heart failure, all-cause mortality, changes in cardiovascular risk factors (e.g., blood pressure, lipid profile, and body weight), and safety outcomes (e.g., severe hypoglycemia, pancreatitis, and thyroid cancer).

Quality Assessment

The quality of the included studies was assessed using the Mixed Method Appraisal Tool (MMAT).

Results

The characteristics of the 15 included studies along with the MMAT sore for quality are given below in Table 2.

**Table 2:** Characteristics of the studies T2D, type 2 diabetes; CV, cardiovascular; ASCVD, atherosclerotic cardiovascular disease; CKD, chronic kidney disease; HF, heart failure; MACE, major adverse cardiovascular events; MASLD, metabolic dysfunction-associated steatotic liver disease; DKD, diabetic kidney disease; RCT, randomized controlled trial

Sr. No.	Author et al. Year	Study type	MMAT score	Objective	Key findings
1	Tan et al., 2023 [21]	Observational cohort study	5	Compare once-weekly GLP-1 RAs vs DPP-4is in T2D patients with ASCVD	GLP-1 RAs are associated with a lower risk of stroke, MI, and reduced healthcare utilization and costs
2	Kutz et al., 2023 [22]	Cohort study	4	Compare cardiovascular effectiveness of SGLT-2is, GLP-1 RAs, and DPP-4is in older T2D patients	SGLT-2is and GLP-1 RAs improved cardiovascular outcomes, with the largest benefits in frail patients
3	Longato et al., 2021 [23]	Retrospective cohort study	4	Compare cardiovascular outcomes of GLP-1 RAs vs. basal insulin in T2D	GLP-1 RA users had better cardiovascular outcomes than basal insulin users
4	Bain et al., 2019 [24]	RCT	5	Assess CV safety of oral semaglutide in T2D patients at high CV risk	Study design and rationale for PIONEER 6 trial
5	Nørgaard et al., 2022 [25]	Cohort study	5	Compare CV outcomes of GLP-1 RAs vs. SGLT-2is in T2D	No significant differences in CV risk between GLP-1 RAs and SGLT-2is
6	Mann et al., 2018 [26]	RCT	5	Evaluate effects of liraglutide on CV events in T2D patients with CKD	Liraglutide reduced the risk of major CV events and all-cause mortality in patients with CKD
7	Fudim et al., 2019 [27]	RCT	4	Assess the effects of exenatide on CV outcomes in T2D patients with/without HF	Exenatide is well-tolerated but benefits are attenuated in patients with baseline HF.
8	Holman et al., 2017 [28]	RCT	4	Evaluate CV effects of once-weekly exenatide in T2D	No significant difference in MACE between exenatide and placebo
9	Xie et al., 2023 [29]	Emulated trial using EHR data	5	Compare the effectiveness of SGLT2is, GLP-1 RAs, DPP-4is, and sulfonylureas on MACE	SGLT2is and GLP-1 RAs are associated with a lower risk of MACE compared to DPP-4is or sulfonylureas.
10	Baviera et al., 2022 [30]	Cohort study	5	Compare effectiveness and safety of GLP-1 RAs vs. SGLT-2is in T2D	GLP-1 RAs are more effective in reducing MACE and MI, equally safe as SGLT-2is
11	Katsuyama et al., 2024 [31]	Retrospective longitudinal study	5	Evaluate effects of once-weekly semaglutide on CV risk factors in Japanese T2D patients	Semaglutide improved body weight, glycemic control, lipid profile, and potentially MASLD
12	Lin et al., 2023 [32]	Cohort study	5	Assess CV and renal effects of GLP-1 RAs in advanced DKD	GLP-1 RAs had a neutral effect on CV outcomes but reduced kidney events in advanced DKD
13	Htoo et al., 2024 [33]	Cohort study	5	Compare cardiorenal effectiveness of empagliflozin vs. GLP-1 RAs	Empagliflozin showed similar or better outcomes for various CV and renal endpoints
14	Dong et al., 2022 [34]	Cohort study	4	Compare CV effectiveness of GLP-1 RAs vs. SGLT2is in T2D	Overall comparable effectiveness, but variations in certain patient subgroups
15	Marso et al., 2016 [35]	RCT	5	Evaluate CV effects of liraglutide in T2D with high CV risk	Liraglutide is associated with lower rates of CV events and death from any cause

Synthesis of GLP-1 RA Effects on Cardiovascular Outcomes

The comprehensive review of 15 studies investigating the effects of GLP-1 RAs on cardiovascular outcomes in patients with T2D revealed a complex landscape of findings. The studies encompassed a range of research designs, including RCTs, observational cohort studies, and retrospective analyses, providing a multifaceted view of the cardiovascular effects of GLP-1 RAs in both clinical trials and real-world settings.

Cardiovascular Outcomes

The majority of studies reported beneficial cardiovascular effects associated with GLP-1 RA use, although the specific outcomes and comparators varied across studies. Tan et al. (2023) conducted an observational cohort study comparing once-weekly GLP-1 RAs to DPP-4 inhibitors in patients with atherosclerotic cardiovascular disease (ASCVD) [21]. Their findings demonstrated a lower risk of stroke and MI in patients treated with GLP-1 RAs. This study not only highlighted the cardiovascular benefits but also reported reduced healthcare utilization and costs associated with GLP-1 RA use, suggesting potential economic advantages alongside clinical benefits.

In a retrospective cohort study, Longato et al. (2021) compared cardiovascular outcomes between GLP-1 RA users and basal insulin users [23]. The results favored GLP-1 RAs, with users experiencing better cardiovascular outcomes compared to those on basal insulin. This finding is particularly noteworthy as it compares GLP-1 RAs to an established diabetes treatment, providing valuable insights for treatment selection in clinical practice.

The LEADER trial, a landmark RCT by Marso et al. (2016), investigated the cardiovascular effects of liraglutide in patients with T2D at high cardiovascular risk [35]. The study reported lower rates of cardiovascular events and death from any cause in patients treated with liraglutide compared to placebo. This trial was instrumental in establishing the cardiovascular benefits of GLP-1 RAs and influenced subsequent clinical guidelines.

While many studies reported positive cardiovascular outcomes, some found neutral or mixed effects. Holman et al. (2017) conducted an RCT evaluating the cardiovascular effects of once-weekly exenatide [28]. Their results showed no significant difference in MACE between the exenatide and placebo groups. This finding highlights the variability in outcomes across different GLP-1 RAs and underscores the importance of considering individual agent effects rather than assuming a uniform class effect.

Nørgaard et al. (2022) compared cardiovascular outcomes between GLP-1 RAs and SGLT-2 inhibitors, another class of diabetes medications with established cardiovascular benefits [25]. Their cohort study found no significant differences in cardiovascular risk between the two classes, suggesting comparable cardiovascular safety profiles. This finding is particularly relevant for clinical decision-making, as it provides insight into the relative effectiveness of two commonly prescribed classes of diabetes medications with cardiovascular benefits.

Comparative Effectiveness

Several studies focused on the comparative effectiveness of GLP-1 RAs against other diabetes medications. Xie et al. (2023) conducted an emulated trial using electronic health record data, comparing the effectiveness of SGLT2 inhibitors, GLP-1 RAs, DPP-4 inhibitors, and sulfonylureas on MACE [29]. Their findings indicated that both SGLT2 inhibitors and GLP-1 RAs were associated with a lower risk of MACE compared to DPP-4 inhibitors or sulfonylureas. This study provides valuable real-world evidence to support the cardiovascular benefits of GLP-1 RAs observed in RCTs.

Baviera et al. (2022) specifically compared the effectiveness and safety of GLP-1 RAs versus SGLT-2 inhibitors in a cohort study [30]. They found that GLP-1 RAs were more effective in reducing MACE and MI while being equally safe as SGLT-2 inhibitors. This direct comparison between two classes of medications with established cardiovascular benefits provides important guidance for clinicians in selecting optimal treatments for patients with T2D and high cardiovascular risk.

Dong et al. (2022) also compared the cardiovascular effectiveness of GLP-1 RAs and SGLT2 inhibitors in a cohort study [34]. While they found overall comparable effectiveness between the two classes, they noted variations in certain patient subgroups. This finding highlights the importance of personalized treatment approaches and the need for further research to identify patient characteristics that may predict better responses to specific medications.

Other Effects and Patient Subgroups

Beyond primary cardiovascular outcomes, several studies investigated the effects of GLP-1 RAs on renal outcomes, metabolic parameters, and in specific patient subgroups. Lin et al. (2023) focused on patients with advanced diabetic kidney disease (DKD) and found that while GLP-1 RAs had a neutral effect on cardiovascular outcomes in this population, they did reduce kidney events [32]. This finding suggests potential renoprotective effects of GLP-1 RAs, even in the absence of clear cardiovascular benefits in this high-risk subgroup.

Katsuyama et al. (2024) evaluated the effects of once-weekly semaglutide on cardiovascular risk factors in Japanese patients with T2D [31]. They observed improvements in body weight, glycemic control, lipid profile, and potentially metabolic dysfunction-associated steatotic liver disease (MASLD). These findings highlight the multifaceted benefits of GLP-1 RAs beyond direct cardiovascular effects, addressing multiple cardiometabolic risk factors simultaneously.

The impact of GLP-1 RAs in specific patient subgroups was also explored. Kutz et al. (2023) found that the cardiovascular benefits of GLP-1 RAs were most pronounced in frail patients, suggesting particular utility in this vulnerable population [22]. Conversely, Fudim et al. (2019) noted that while exenatide was well-tolerated in patients with heart failure, its benefits were attenuated in this subgroup compared to those without baseline heart failure [27]. This finding underscores the importance of considering comorbidities when selecting diabetes treatments with cardiovascular effects.

Thus, the synthesis of these 15 studies provides a comprehensive overview of the cardiovascular effects of GLP-1 RAs in patients with T2D. While the majority of studies reported positive cardiovascular outcomes, the variability in findings across different GLP-1 RAs, patient populations, and comparator treatments highlights the complexity of this therapeutic area. The evidence suggests that GLP-1 RAs offer cardiovascular benefits for many patients with T2D, particularly those at high cardiovascular risk. However, the effectiveness may vary depending on the specific agent, patient characteristics, and comorbidities.

The comparative effectiveness studies position GLP-1 RAs favorably against other diabetes medications, including DPP-4 inhibitors and, in some cases, SGLT-2 inhibitors. The additional benefits observed in renal outcomes, metabolic parameters, and healthcare utilization further support the role of GLP-1 RAs in comprehensive diabetes management. The summary of the effects of GLP-1 RA is given in Table 3.

**Table 3:** Summary of GLP-1 RA effects CV, cardiovascular; MI, myocardial infarction; MACE, major adverse cardiovascular events; CKD, chronic kidney disease; HF, heart failure; MASLD, metabolic dysfunction-associated steatotic liver disease; DKD, diabetic kidney disease; GLP-1 RAs, glucagon-like peptide-1 receptor agonists

GLP-1 RA type	Key effects	Studies
Once-weekly GLP-1 RAs	Lower the risk of stroke, MI, and reduced healthcare utilization and costs compared to DPP-4is	Tan et al., 2023 [21]
GLP-1 RAs (not specified)	Improved cardiovascular outcomes, with the largest benefits in frail patients	Kutz et al., 2023 [22]
GLP-1 RAs (not specified)	Better cardiovascular outcomes compared to basal insulin	Longato et al., 2021 [23]
Oral semaglutide	Study design only; results not reported	Bain et al., 2019 [24]
GLP-1 RAs (not specified)	No significant differences in CV risk compared to SGLT-2is	Nørgaard et al., 2022 [25]
Liraglutide	Reduced risk of major CV events and all-cause mortality in patients with CKD	Mann et al., 2018 [26]
Exenatide (once-weekly)	Well-tolerated but benefits attenuated in patients with baseline HF	Fudim et al., 2019 [27]
Exenatide (once-weekly)	No significant difference in MACE compared to the placebo	Holman et al., 2017 [28]
GLP-1 RAs (not specified)	Lower risk of MACE compared to DPP-4is or sulfonylureas	Xie et al., 2023 [29]
GLP-1 RAs (not specified)	More effective in reducing MACE and MI compared to SGLT-2is	Baviera et al., 2022 [30]
Semaglutide (once-weekly)	Improved body weight, glycemic control, lipid profile, and potentially MASLD	Katsuyama et al., 2024 [31]
GLP-1 RAs (not specified)	Neutral effect on CV outcomes but reduced kidney events in advanced DKD	Lin et al., 2023 [32]
GLP-1 RAs (cardioprotective)	Similar or worse outcomes for various CV and renal endpoints compared to empagliflozin	Htoo et al., 2024 [33]
GLP-1 RAs (not specified)	Comparable CV effectiveness to SGLT2is overall, with variations in certain subgroups	Dong et al., 2022 [34]
Liraglutide	Lower rates of CV events and death from any cause compared to placebo	Marso et al., 2016 [35]

Discussion

The results of this systematic review provide compelling evidence for the cardiovascular benefits of GLP-1 RAs in patients with T2D. These findings align with and expand upon previous research, offering new insights into the comparative effectiveness of GLP-1 RAs against other diabetes medications and their impact on various patient subgroups.

Our review consistently demonstrates the positive impact of GLP-1 RAs on cardiovascular outcomes, particularly in reducing the risk of MACE, stroke, and MI. These findings are in line with large-scale CVOTs such as LEADER (Marsoet al., 2016) and REWIND (Gerstein et al., 2019), which showed significant cardiovascular benefits with liraglutide and dulaglutide, respectively [12,35]. The consistency of these results across different study designs and populations strengthens the evidence for the cardioprotective effects of GLP-1 RAs.

The comparative effectiveness studies in our review (e.g., Tan et al., 2023; Xie et al., 2023) suggest that GLP-1 RAs may offer superior cardiovascular protection compared to DPP-4 inhibitors and sulfonylureas [21,29]. This is consistent with a network meta-analysis by Palmer et al. (2021), which found GLP-1 RAs to be more effective in reducing cardiovascular mortality than DPP-4 inhibitors [36]. These findings have important implications for treatment selection in clinical practice, particularly for patients with T2D and high cardiovascular risk.

Interestingly, our review includes studies comparing GLP-1 RAs to SGLT-2 inhibitors, another class of diabetes medications with established cardiovascular benefits. While some studies (e.g., Nørgaard et al., 2022) found no significant differences between the two classes, others (Baviera et al., 2022) suggested potential advantages of GLP-1 RAs in certain outcomes [25,30]. This aligns with findings from the AMPLITUDE-O trial (Sattar et al., 2021), which demonstrated cardiovascular benefits of the GLP-1 RA efpeglenatide in patients already on SGLT-2 inhibitors, suggesting potential additive effects [13]. The relative efficacy of GLP-1 RAs and SGLT-2 inhibitors remains an area of active research and debate, with implications for treatment algorithms and personalized medicine approaches.

Furthermore, the mechanisms underlying the cardiovascular benefits of GLP-1 RAs are multifaceted and not fully elucidated. Nauck et al. (2017) proposed both direct and indirect effects on the cardiovascular system [15]. Direct effects may include improvements in endothelial function, reduction of inflammation, and modulation of cardiac metabolism. Indirect effects are mediated through improvements in glycemic control, body weight reduction, and favorable changes in lipid profiles [15].

Recent research has also suggested potential anti-atherosclerotic effects of GLP-1 RAs. A study by Rakipovski et al. (2018) demonstrated that liraglutide and semaglutide reduced atherosclerosis in mouse models through mechanisms involving inflammatory pathways [16]. These findings provide a potential explanation for the reduced risk of atherosclerotic events observed in clinical trials and real-world studies.

Additionally, the renal benefits observed in some studies (e.g., Lin et al., 2023) are consistent with previous research [32]. A meta-analysis by Kristensen et al. (2019) found that GLP-1 RAs reduced the risk of kidney disease progression in patients with T2D [17]. However, the varying effects across different stages of kidney disease highlighted in our review suggest a need for a more nuanced understanding of GLP-1 RA effects in specific renal subpopulations.

The potential renoprotective effects of GLP-1 RAs are particularly important given the high prevalence of chronic kidney disease (CKD) in patients with T2D. Muskiet et al. (2020) proposed several mechanisms for the renal benefits of GLP-1 RAs, including reduction of renal inflammation, improvement of renal hemodynamics, and direct effects on proximal tubular cells [37]. These findings suggest that GLP-1 RAs may offer a dual cardio-renal protective effect, which could be particularly beneficial for patients with T2D and CKD.

Regarding the metabolic effects, the improvements in body weight, glycemic control, and lipid profile noted in our review (e.g., Katsuyama et al., 2024) are well-established effects of GLP-1 RAs [31]. A systematic review by Aroda (2018) similarly reported consistent weight loss and improved glycemic control with GLP-1 RAs [38]. The potential benefits of MASLD align with emerging research, such as the LEAN study (Armstrong et al., 2016), which showed promising effects of liraglutide in non-alcoholic steatohepatitis [39].

The metabolic effects of GLP-1 RAs may contribute to their cardiovascular benefits through multiple pathways. Weight loss and improved glycemic control can lead to reduced insulin resistance, improved lipid profiles, and decreased systemic inflammation, all of which are risk factors for CVD. Moreover, the potential benefits of MASLD are particularly intriguing given the growing recognition of the link between liver disease and cardiovascular risk in patients with T2D [40].

Our review highlights the importance of considering patient characteristics in GLP-1 RA treatment. The observation of larger benefits in frail patients (Kutz et al., 2023) is particularly noteworthy and aligns with findings from a post-hoc analysis of the LEADER trial (Gilbert et al., 2019), which found that older, frail patients derived similar cardiovascular benefits from liraglutide as their younger counterparts [22,41]. This suggests that GLP-1 RAs may be particularly valuable in vulnerable patient populations, where the need for cardiovascular protection is often greatest.

The attenuated benefits in patients with baseline heart failure (Fudim et al., 2019) raise important questions about the optimal use of GLP-1 RAs in this population [27]. This finding contrasts with some studies, such as the LIVE trial (Jorsal et al., 2017), which found no increased risk of heart failure with liraglutide treatment [42]. The findings of our review have several important implications for clinical practice. First, they support the use of GLP-1 RAs as a preferred treatment option for patients with T2D and established CVD or high cardiovascular risk, in line with current guidelines from major diabetes organizations [3].

The relationship between GLP-1 RAs and heart failure remains complex, with some studies suggesting potential benefits (Margulies et al., 2016) and others showing neutral effects (Jorsal et al., 2017) [42,43]. Moreover, the benefits observed in frail patients and the potential renoprotective effects suggest that GLP-1 RAs may be particularly valuable in older patients and those with CKD, two populations that often present challenges in diabetes management. Rigorous research is needed to clarify the effects of GLP-1 RAs in heart failure patients and to identify which subgroups of heart failure patients might benefit most from these agents.

Finally, the comparative effectiveness data suggest that GLP-1 RAs may be preferred over DPP-4 inhibitors or sulfonylureas in patients requiring intensification of glucose-lowering therapy, particularly if cardiovascular protection is a priority. The potential additive effects of SGLT-2 inhibitors also raise the possibility of combination therapy for maximum cardio-renal protection in high-risk patients.

Limitations

Several limitations should be considered when interpreting these results, as the heterogeneity in study designs, populations, and specific GLP-1 RAs used may limit direct comparisons across studies. The observational nature of some included studies introduces the potential for confounding and bias. Furthermore, the long-term effects beyond the study durations are not captured, necessitating further research on the sustained benefits of GLP-1 RAs. Patient-reported outcomes and quality-of-life measures were not consistently reported across studies.

Future recommendations

Based on the findings of this review and current gaps in knowledge, we recommend head-to-head trials comparing different GLP-1 RAs to elucidate potential differences within the class. Additionally, studies should focus on the effects of GLP-1 RAs in specific subpopulations, particularly those with heart failure or advanced kidney disease. Research into combination therapies, especially GLP-1 RA and SGLT-2 inhibitor combinations, is also necessary to explore potential synergistic effects. Furthermore, long-term follow-up studies should be considered to assess the durability of cardiovascular and renal benefits. Investigating the cost-effectiveness of GLP-1 RAs across different healthcare systems would inform policy decisions. Finally, further research is needed to explore the mechanisms underlying the cardiovascular benefits of GLP-1 RAs, including potential direct effects on the cardiovascular system.

## Conclusions

This systematic review demonstrates that GLP-1 receptor agonists effectively reduce cardiovascular risk in patients with T2D, particularly for MACE, stroke, and MI. The benefits extend beyond cardiovascular outcomes to include improvements in renal outcomes and metabolic parameters. While these agents show promise as preferred treatment options for patients with established CVD or high cardiovascular risk, their effectiveness may vary based on specific patient characteristics and comorbidities, highlighting the importance of personalized treatment approaches.

## References

[1] Khan MA, Hashim MJ, King JK, Govender RD, Mustafa H, Al Kaabi J (2020). Epidemiology of type 2 diabetes - global burden of disease and forecasted trends. J Epidemiol Glob Health.

[2] ElSayed NA, Aleppo G, Aroda VR (2023). 2. Classification and diagnosis of diabetes: standards of care in diabetes-2023. Diabetes Care.

[3] Davies MJ, D'Alessio DA, Fradkin J (2018). Management of hyperglycemia in type 2 diabetes, 2018. A consensus report by the American Diabetes Association (ADA) and the European Association for the Study of Diabetes (EASD). Diabetes Care.

[4] Blonde L, Umpierrez GE, Reddy SS (2022). American Association of Clinical Endocrinology clinical practice guideline: developing a diabetes mellitus comprehensive care plan—2022 update. Endocr Pract.

[5] Nauck MA, Meier JJ (2018). Incretin hormones: their role in health and disease. Diabetes Obes Metab.

[6] Drucker DJ (2018). Mechanisms of action and therapeutic application of glucagon-like peptide-1. Cell Metab.

[7] Baviera M, Genovese S, Lepore V (2021). Lower risk of death and cardiovascular events in patients with diabetes initiating glucagon-like peptide-1 receptor agonists or sodium-glucose cotransporter-2 inhibitors: a real-world study in two Italian cohorts. Diabetes Obes Metab.

[8] Nissen SE, Wolski K (2007). Effect of rosiglitazone on the risk of myocardial infarction and death from cardiovascular causes. N Engl J Med.

[9] US Food (2008). Drug administration guidance for industry diabetes mellitus—evaluating cardiovascular risk in new antidiabetic therapies to treat type 2 diabetes. Diabetes.

[10] Cefalu WT, Kaul S, Gerstein HC (2018). Cardiovascular outcomes trials in type 2 diabetes: where do we go from here? Reflections from a diabetes care editors’ Expert Forum. Diabetes Care.

[11] Gerstein HC, Colhoun HM, Dagenais GR (2019). Dulaglutide and cardiovascular outcomes in type 2 diabetes (REWIND): a double-blind, randomised placebo-controlled trial. Lancet.

[12] Gerstein HC, Sattar N, Rosenstock J (2021). Cardiovascular and renal outcomes with Efpeglenatide in type 2 diabetes. N Engl J Med.

[13] Sattar N, Lee MM, Kristensen SL (2021). Cardiovascular, mortality, and kidney outcomes with GLP-1 receptor agonists in patients with type 2 diabetes: a systematic review and meta-analysis of randomised trials. Lancet Diabetes Endocrinol.

[14] Rangaswami J, Bhalla V, de Boer IH (2020). Cardiorenal protection with the newer antidiabetic agents in patients with diabetes and chronic kidney disease: a scientific statement from the American Heart Association. Circulation.

[15] Nauck MA, Meier JJ, Cavender MA, Abd El Aziz M, Drucker DJ (2017). Cardiovascular actions and clinical outcomes with glucagon-like peptide-1 receptor agonists and dipeptidyl peptidase-4 inhibitors. Circulation.

[16] Rakipovski G, Rolin B, Nøhr J (2018). The GLP-1 analogs liraglutide and semaglutide reduce atherosclerosis in ApoE(-/-) and LDLR(-/-) mice by a mechanism that includes inflammatory pathways. JACC Basic Transl Sci.

[17] Kristensen SL, Rørth R, Jhund PS (2019). Cardiovascular, mortality, and kidney outcomes with GLP-1 receptor agonists in patients with type 2 diabetes: a systematic review and meta-analysis of cardiovascular outcome trials. Lancet Diabetes Endocrinol.

[18] Tsapas A, Karagiannis T, Avgerinos I, Matthews DR, Bekiari E (2021). Comparative effectiveness of glucose-lowering drugs for type 2 diabetes. Ann Intern Med.

[19] Zelniker TA, Wiviott SD, Raz I (2019). Comparison of the effects of glucagon-like peptide receptor agonists and sodium-glucose cotransporter 2 inhibitors for prevention of major adverse cardiovascular and renal outcomes in type 2 diabetes mellitus. Circulation.

[20] Schernthaner G, Shehadeh N, Ametov AS (2020). Worldwide inertia to the use of cardiorenal protective glucose-lowering drugs (SGLT2i and GLP-1 RA) in high-risk patients with type 2 diabetes. Cardiovasc Diabetol.

[21] Tan X, Liang Y, Rajpura JR (2023). Once-weekly glucagon-like peptide-1 receptor agonists vs dipeptidyl peptidase-4 inhibitors: cardiovascular effects in people with diabetes and cardiovascular disease. Cardiovasc Diabetol.

[22] Kutz A, Kim DH, Wexler DJ, Liu J, Schneeweiss S, Glynn RJ, Patorno E (2023). Comparative cardiovascular effectiveness and safety of SGLT-2 inhibitors, GLP-1 receptor agonists, and DPP-4 inhibitors according to frailty in type 2 diabetes. Diabetes Care.

[23] Longato E, Di Camillo B, Sparacino G, Tramontan L, Avogaro A, Fadini GP (2021). Cardiovascular outcomes after initiating GLP-1 receptor agonist or basal insulin for the routine treatment of type 2 diabetes: a region-wide retrospective study. Cardiovasc Diabetol.

[24] Bain SC, Mosenzon O, Arechavaleta R (2019). Cardiovascular safety of oral semaglutide in patients with type 2 diabetes: rationale, design and patient baseline characteristics for the PIONEER 6 trial. Diabetes Obes Metab.

[25] Nørgaard CH, Starkopf L, Gerds TA (2022). Cardiovascular outcomes with GLP-1 receptor agonists vs. SGLT-2 inhibitors in patients with type 2 diabetes. Eur Heart J Cardiovasc Pharmacother.

[26] Mann JF, Fonseca V, Mosenzon O (2018). Effects of liraglutide versus placebo on cardiovascular events in patients with type 2 diabetes mellitus and chronic kidney disease. Circulation.

[27] Fudim M, White J, Pagidipati NJ (2019). Effect of once-weekly exenatide in patients with type 2 diabetes mellitus with and without heart failure and heart failure-related outcomes: insights from the EXSCEL trial. Circulation.

[28] Holman RR, Bethel MA, Mentz RJ (2017). Effects of once-weekly exenatide on cardiovascular outcomes in type 2 diabetes. N Engl J Med.

[29] Xie Y, Bowe B, Xian H, Loux T, McGill JB, Al-Aly Z (2023). Comparative effectiveness of SGLT2 inhibitors, GLP-1 receptor agonists, DPP-4 inhibitors, and sulfonylureas on risk of major adverse cardiovascular events: emulation of a randomised target trial using electronic health records. Lancet Diabetes Endocrinol.

[30] Baviera M, Foresta A, Colacioppo P (2022). Effectiveness and safety of GLP-1 receptor agonists versus SGLT-2 inhibitors in type 2 diabetes: an Italian cohort study. Cardiovasc Diabetol.

[31] Katsuyama H, Hakoshima M, Kaji E (2024). Effects of once-weekly semaglutide on cardiovascular risk factors and metabolic dysfunction-associated steatotic liver disease in Japanese patients with type 2 diabetes: a retrospective longitudinal study based on real-world data. Biomedicines.

[32] Lin Y, Wang TH, Tsai ML (2023). The cardiovascular and renal effects of glucagon-like peptide 1 receptor agonists in patients with advanced diabetic kidney disease. Cardiovasc Diabetol.

[33] Htoo PT, Tesfaye H, Schneeweiss S (2024). Cardiorenal effectiveness of empagliflozin vs. glucagon-like peptide-1 receptor agonists: final-year results from the EMPRISE study. Cardiovasc Diabetol.

[34] Dong YH, Chang CH, Lin JW, Yang WS, Wu LC, Toh S (2022). Comparative cardiovascular effectiveness of glucagon-like peptide-1 receptor agonists versus sodium-glucose cotransporter-2 inhibitors in patients with type 2 diabetes: a population-based cohort study. Diabetes Obes Metab.

[35] Marso SP, Daniels GH, Brown-Frandsen K (2016). Liraglutide and cardiovascular outcomes in type 2 diabetes. N Engl J Med.

[36] Palmer SC, Tendal B, Mustafa RA (2021). Sodium-glucose cotransporter protein-2 (SGLT-2) inhibitors and glucagon-like peptide-1 (GLP-1) receptor agonists for type 2 diabetes: systematic review and network meta-analysis of randomised controlled trials. BMJ.

[37] Muskiet MH, Tonneijck L, Smits MM (2017). GLP-1 and the kidney: from physiology to pharmacology and outcomes in diabetes. Nat Rev Nephrol.

[38] Aroda VR (2018). A review of GLP-1 receptor agonists: evolution and advancement, through the lens of randomised controlled trials. Diabetes Obes Metab.

[39] Armstrong MJ, Gaunt P, Aithal GP (2016). Liraglutide safety and efficacy in patients with non-alcoholic steatohepatitis (LEAN): a multicentre, double-blind, randomised, placebo-controlled phase 2 study. Lancet.

[40] Targher G, Tilg H, Byrne CD (2021). Non-alcoholic fatty liver disease: a multisystem disease requiring a multidisciplinary and holistic approach. Lancet Gastroenterol Hepatol.

[41] Gilbert MP, Bain SC, Franek E (2019). Effect of liraglutide on cardiovascular outcomes in elderly patients: a post hoc analysis of a randomized controlled trial. Ann Intern Med.

[42] Jorsal A, Kistorp C, Holmager P (2017). Effect of liraglutide, a glucagon-like peptide-1 analogue, on left ventricular function in stable chronic heart failure patients with and without diabetes (LIVE)-a multicentre, double-blind, randomised, placebo-controlled trial. Eur J Heart Fail.

[43] Margulies KB, Hernandez AF, Redfield MM (2016). Effects of Liraglutide on Clinical Stability Among Patients With Advanced Heart Failure and Reduced Ejection Fraction: A Randomized Clinical Trial. J Am Med Assoc.

